# Development and validation of a Chinese medication literacy measure

**DOI:** 10.1111/hex.12569

**Published:** 2017-05-05

**Authors:** Ying‐Chih Yeh, Hsiang‐Wen Lin, Elizabeth H. Chang, Yen‐Ming Huang, Yu‐Chieh Chen, Chun‐Yu Wang, Jen‐Wei Liu, Yu Ko

**Affiliations:** ^1^ School of Pharmacy College of Pharmacy Taipei Medical University Taipei Taiwan; ^2^ School of Pharmacy and Graduate Institute China Medical University Taichung Taiwan; ^3^ Research Center for Pharmacoeconomics College of Pharmacy Taipei Medical University Taipei Taiwan; ^4^ Division of Social and Administrative Sciences School of Pharmacy University of Wisconsin‐Madison Madison WI USA; ^5^ Department of Pharmacy China Medical University Hospital Taichung Taiwan; ^6^ Taiwan Society of Health‐System Pharmacists Taipei Taiwan; ^7^ Department of Pharmacy Shin Kong Wu Ho‐Su Memorial Hospital Taipei Taiwan; ^8^ Department of Pharmacy Wan Fang Hospital Taipei Medical University Taipei Taiwan

**Keywords:** health literacy, medication literacy, Taiwan, validation

## Abstract

**Background:**

Despite the impact of medication literacy (ML) on patients’ safe use of medications, existing instruments are mostly for general health literacy measurement or designed for specific disease populations, with few specifically designed for ML.

**Objective:**

To develop and validate the first Chinese medication literacy measure (ChMLM).

**Methods:**

The ChMLM was developed by a multidisciplinary and bilingual expert panel and subsequently pilot‐tested. The final version had 17 questions in four sections: vocabulary, non‐prescription drug, prescription drug and drug advertisement. Face‐to‐face interviews were administered in a convenience sample of adults with diverse sociodemographic characteristics. Internal consistency was assessed by Cronbach's alpha. Content validity was confirmed by the expert panel, and hypothesis testing was performed to assess construct validity.

**Results:**

A total of 634 adults were interviewed. The mean (SD) total ChMLM score was 13.0 (2.8). The internal validity was acceptable (Cronbach's alpha=0.72). Nine of the ten a priori hypotheses were fulfilled. Younger age, higher income and higher education levels were significantly associated with a higher ChMLM score. Furthermore, higher scores on the ChMLM were associated with higher confidence or less difficulty in writing, reading, speaking and listening abilities in a health‐care encounter. No association was found between ChMLM total scores and frequency of doctor's visits.

**Conclusion:**

The ChMLM is a valid and reliable ML measure. It may help pharmacists and other health‐care providers to target patients and problem areas that need interventions with the ultimate goal of preventing medication errors and harm.

## 1 INTRODUCTION

Health literacy (HL) is defined as the degree to which individuals have the capacity to obtain, process and understand basic health information and services needed to make appropriate health decisions.^1^ Health literacy has increasingly gained interest in the field of public health and health care because of its influence on patients’ health. Low HL is found to be associated with adverse health outcomes, including higher risk of emergency care use^2^, ^3^, ^4^, ^5^, ^6^, ^7^ and hospitalization,^3^, ^7^, ^8^ poorer overall health status^3^, ^9^, ^10^, ^11^, ^12^, ^13^ and higher mortality rate.^9^, ^14^, ^15^

In addition to general health outcomes, a few studies have found an association between inadequate HL and medication‐related skills, including dosing errors,^16^ misunderstanding of prescription labels,^17^ poorer ability to take medication appropriately,^17^, ^18^, ^19^, ^20^, ^21^, ^22^, ^23^, ^24^, ^25^, ^26^, ^27^ use of non‐standardized dosing tools^27^ and lack of knowledge of weight‐based dosing.^27^ Although medication literacy (ML), or the ability to read, understand and process medication‐related information, is assumed to be related to HL, ML may not be fully and adequately captured by general HL assessments. A lack of adequate ML could result in poor medication adherence and the misunderstanding of medication‐related information or instructions, which could in turn make patients more prone to medication errors that adversely affect their health.

Despite the impact of ML on patients’ safe use of medications, existing instruments are mostly for general HL measurement or designed for specific disease populations, with few specifically designed for ML. Stilley et al.^28^ developed a medication health literacy screen as a measure of use and understanding of information on prescription labels. The instrument contained two labels: one for an immunosuppressant medication and one for a diabetes medication. Another ML measure, the Medication Literacy Assessment in Spanish and English (MedLitRxSE), developed by Sauceda et al.,^29^ aimed to assess individuals’ ability to access, understand and act on medication information. Three interrelated constructs were tested in this instrument, namely prose literacy, document literacy and numeracy. Both instruments have demonstrated good psychometric properties.

To assess individuals’ ML levels and subsequently create a supportive environment that encourages correct use of medications, it is essential to develop a valid and reliable ML tool. The main goal of this study was to develop and validate the first Chinese ML measure for the general adult population in Taiwan.

## 2 METHODS

### 2.1 Instrument development

The initial list of ChMLM items was generated by a pharmacist researcher (HWL) adapting previously validated medication‐related instruments found in the literature.^28^, ^29^, ^30^, ^31^, ^32^, ^33^ The first version of the instrument, which contained 25 items, was reviewed by a panel of 11 multidisciplinary and bilingual experts, including pharmacists, health literacy experts and researchers with backgrounds in psychometrics, communication, education, health literacy and clinical pharmacy. Half of the experts also had experience in the translation and validation of patient‐reported measures. The experts were asked to rate each item's importance and appropriateness/relevance on a 5‐point scale, ranging from “not important/adequate at all” to “very important/adequate.” In addition, the experts were encouraged to modify and/or comment on the items and explain their rationales. Several iterations of feedback and discussion among the experts generated the revised version. Pilot testing was performed in a convenience sample of 35 individuals with diverse demographic characteristics. These participants were asked to identify ambiguous or unclear questions and suggest an alternative wording. As a result, minor changes were made to enhance clarity and comprehension.

The final version of the ChMLM consisted of seventeen items divided into four sections: vocabulary (five questions), non‐prescription drug (five questions), prescription drug (four questions) and drug advertisement (three items). Each section involved different medication props. Section one evaluated the respondent's ability to interpret a list of five medication‐related words/phrases (ie, dose, ingredient, combination drug, external use and side‐effect). Section two tested the respondent's ability to read a mock package and insert for a non‐prescription medication (i.e patient information sheet) to find the correct information about the drug's indications, warnings, dosing directions, expiration date and the total number of pills contained in the box. Section three assessed the respondent's ability to read the written information on the carrier of a fabricated diabetic medication and correctly answer questions about the drug's next dosing time, side‐effects and total number of days prescribed. In addition, there was an item that tested whether the respondent could tell the difference between two prescription labels. Section four asked the respondent to evaluate an exaggerated drug advertisement and find the information about the drug's indication and side‐effect. Section one had true/false questions, whereas all the questions in sections two through four were multiple‐choice questions with four response options, including “I don't know/I'm not sure.” The medication props used for sections two and three had similar format and looked like actual drug products. The ChMLM was intended to assess the skills an individual would need in various real‐world scenarios, including interpreting medication terms, comprehending medication instructions and calculating dosing intervals.

### 2.2 Study setting and participants

The final version of the ChMLM was administered through face‐to‐face interviews in a convenience sample of the general population in Taiwan from September 2015 to November 2015. Thirty‐four pharmacy undergraduate students and research assistants were trained as interviewers by standardized procedures and multiple rehearsals. The interviewers were reminded to be non‐judgemental, avoid overinterpreting the questions and encourage interviewees to try their best on the test and avoid guessing. The interviewers’ friends, relatives, neighbours and the customers/members of participating pharmacies and organizations were approached as potential participants. Potential participants were referred by the pharmacists in the participating community pharmacies, and the interviews were conducted in front of the pharmacies or in nearby areas. The participants were not only necessarily customers of the pharmacies but also included community residents who were acquaintances of the pharmacists. To be eligible, participants needed to be at least 20 years old and able to speak Mandarin or Taiwanese. Exclusion criteria were having speaking, hearing or cognitive impairment that precluded the participants from adequately interacting with the interviewer. The questionnaire was placed on the Internet by Survey Monkey, and the participants’ responses were collected either directly using an electronic device (eg, mobile phone, iPad) or indirectly via a hard copy of the questionnaire that was later transferred to the online version by the interviewer. Participants were offered a choice of self‐administration or verbal administration by the interviewer. There was no set time limit for completion of the interview, and each participant was given an NTD$50 convenience store voucher after completing the interview. The study was approved by the institutional review board of the China Medical University & Hospital Research Ethics Center.

### 2.3 Interview instrument

In addition to the ChMLM items, the interview instrument also collected respondents’ sociodemographic information, such as age, gender, education level and personal annual income. For validation purposes, we also asked respondents how frequently they visit a doctor and to self‐report their confidence or difficulty in writing, reading, speaking and listening abilities in a health‐care encounter. Specifically, we asked how confident they were when filling out medical forms in hospitals, how confident they were in their ability to read and understand written information provided by hospitals, how difficult it was to ask health providers medication‐related questions and how difficult it was to understand health‐care providers’ verbal explanations. These health literacy screening questions have been validated in previous studies.^34^, ^35^, ^36^, ^37^ These items used a 5‐point response scale ranging from very confident/never have difficulty to not confident at all/always have difficulty.

### 2.4 Psychometric evaluation

The ChMLM total score was calculated by the number of questions answered correctly (a score of 1 was assigned for each correct answer). Several psychometric properties of the ChMLM were assessed, including the percentage of correct responses and the correlation between each item and the total score. Internal consistency was tested by Cronbach's alpha. A Cronbach's alpha coefficient equal to or larger than 0.70 is considered acceptable.^38^ Content validity was confirmed by the expert panel. Factor analysis and hypothesis testing were performed to assess the construct validity of the scale. Spearman's correlation was used to test a priori hypotheses that a higher ChMLM total score is associated with higher education levels, higher income, higher frequency of doctor visit and respondents’ self‐reported higher confidence or less difficulty in their writing, reading, speaking and listening abilities in a health‐care encounter. One‐way ANOVA with Scheffe's test was performed to test the hypotheses that a higher total score was associated with living in the northern residential areas of Taiwan (ie, the most urbanized and populous metropolitan area in Taiwan) and with speaking Mandarin more commonly. In addition, Pearson's correlation analysis was used to examine the association between age and performance on the ChMLM.

All analyses were performed using PASW Statistics 18 (PASW Statistics for Windows, SPSS Inc., Chicago, IL, USA). The level of significance was set at probability (*P*)<.05.

## 3 RESULTS

A total of 634 eligible adults with diverse sociodemographic characteristics gave their consent and were enrolled. Among the 634 enrolled participants, 95.0% (N=602) completed all ChMLM questions, and their responses were included in the validity and reliability analyses. The sociodemographic characteristics of these respondents are summarized in Table 1. The respondents were mostly female (63.6%) and had attended college or graduate school (63.3%). Slightly less than half of the respondents’ annual personal income was <150,000NTD (46.3%), and about the same percentage spoke both Chinese and Taiwanese frequently (47.7%). The mean age of the respondents was 42.2 (SD=16.5) years.

**Table 1:** Sociodemographic characteristics of the survey respondents (N=602)

Characteristics	n (%)^a^
Gender	
Male	218 (36.2)
Female	383 (63.6)
Education level	
Elementary school	33 (5.5)
Junior high	35 (5.8)
Senior high	150 (24.9)
College	334 (55.5)
Graduate school	47 (7.8)
Others	2 (0.3)
Language most commonly spoken	
Mandarin and Taiwanese	287 (47.7)
Mandarin only	276 (45.8)
Taiwanese only	36 (6.0)
Occupation	
Service	124 (20.6)
Student	108 (17.9)
Housekeeper	86 (14.3)
Manufacturing	63 (10.5)
Military, government, or education	44 (7.3)
Finance	39 (6.5)
Freelance	34 (5.6)
Health care	20 (3.3)
Information technology	11 (1.8)
Others	70 (11.6)
Residential areas	
Northern	199 (33.1)
Central northern	61 (10.1)
Central	166 (27.6)
Central‐southern	59 (9.8)
Southern	115 (19.1)
Eastern	1 (0.2)
Personal annual income (in NTD)^b^	
<150,000	279 (46.3)
150 001‐300 000	68 (11.3)
300 001‐450 000	76 (12.6)
450 001‐600 000	78 (13.0)
>600 000	99 (16.4)
Doctor visit frequency in past 3 mo	
Several times a week	10 (1.7)
Once a week	26 (4.3)
Once a month	128 (21.3)
Once	232 (38.5)
None	204 (33.9)
Currently taking medicine	
No	391 (65.0)
Yes	208 (34.6)

a Percentages may not total 100% due to missing data.

b In 2015, 1 NTD=0.031 USD, and the mean personal annual income was 623,535NTD.

**Figure 1:**
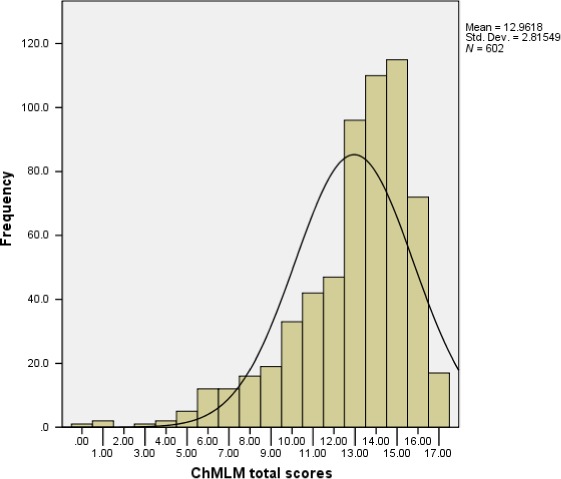
Distribution of the Chinese medication literacy measure total scores

The total number of ChMLM items answered correctly ranged from 0 to 17, with a mean±SD of 13.0±2.8 (Figure 1). The scores were negatively skewed, but the ceiling effect was not evident (2.8% had a full score). The internal consistency of the ChMLM was acceptable with a Cronbach's alpha value of 0.72. As shown in Table 2, the items’ percentage of correct responses ranged from 28.7% to 93.9%. Among the seventeen items, fourteen had item‐total correlations of 0.40 or higher. Principal component analysis showed that the first factor accounted for 20.6% of the total variance and had an eigenvalue of 3.51, which was significantly higher than that of the second component (eigenvalue=1.55) and those of the subsequent components. Moreover, all items had a loading value greater than 0.3 on the first component except for one item in the vocabulary section (factor loading=0.24) and one in the drug advertisement section (factor loading=0.17). The items with the highest factor loadings were those related to the non‐prescription drug label and insert.

**Table 2:** Psychometric properties of the individual items

	% Correct	Item‐total correlation	Factor loading
Vocabulary			
External use	86.4	0.31	0.24
Combination drug	44.9	0.41	0.34
Dose	85.4	0.43	0.38
Side‐effect	79.6	0.48	0.45
Ingredient	78.1	0.48	0.46
Non‐prescription drug			
Indication	90.9	0.49	0.55
Dosing directions	93.0	0.52	0.62
Number of tablets contained	87.7	0.52	0.59
Expiration date	83.4	0.52	0.59
Warning	71.3	0.56	0.58
Rx drug			
Time to take next dose	88.9	0.32	0.31
Number of tablets prescribed	93.9	0.41	0.49
Symptoms of hypoglycaemia	72.1	0.41	0.38
Wrong prescription	52.2	0.43	0.38
Drug advertisement			
Indication	90.7	0.41	0.44
Information accuracy assessment	69.3	0.50	0.47
Side‐effect	28.7	0.27	0.17

Nine of the ten hypotheses were fulfilled. When interpreting the results of an association analysis, one examines the correlation coefficient, which will range from −1 to 1; the greater the absolute value, the stronger the association. A positive correlation coefficient indicates positive correlation, whereas a negative value indicates negative correlation. Respondents with a higher ChMLM total score were younger (r=−0.42; *P* <.001) and with higher income (r_s_=0.14; *P*=.001) and higher education levels (r_s_=0.43; *P*<.001). In addition, higher scores on the ChMLM were associated with higher confidence in the ability to fill out medical forms in hospitals (r_s_=0.25; *P*<.001), higher confidence in the ability to read and understand written information provided by hospitals (r_s_=0.21; *P*<.001), less difficulty understanding health‐care providers’ verbal explanations (r_s_=−0.15; *P*<.001) and less difficulty asking health providers medication‐related questions (r_s_=−0.14; *P*=.001). ANOVA with post hoc comparison results indicated that the respondents in northern residential areas had a higher ChMLM total score than those in the southern residential areas. Moreover, the mean ChMLM total scores were the highest in those who more commonly spoke Mandarin, followed by those who spoke both Mandarin and Taiwanese and then those who spoke Taiwanese more commonly (*P*<.001). Nevertheless, there was no association between ChMLM total scores and frequency of doctor visit (r_s_=0.03; *P*=.53).

## 4 DISCUSSION

In this study, an ML instrument, the ChMLM, was developed to assess an individual's ability to interpret medication‐related vocabulary, read and comprehend prescription and non‐prescription drug instructions and evaluate a drug advertisement. Analysis results demonstrated that the ChMLM possesses good internal consistency, content validity and construct validity.

Unlike word‐recognition HL tools such as the Rapid Estimate of Adult Literacy in Medicine (REALM), the ChMLM incorporates a variety of medication encounters (eg, vocabulary, labels, advertisement). Moreover, it is one of the few tools that focus on medication literacy, as most existing validated instruments are for general HL or disease‐specific literacy. The psychometric evaluation of the ChMLM produced generally satisfactory results; however, an item that asked about an advertised product's side‐effect had both a low item‐total correlation value and a low factor loading. The relatively low correct rate of this item indicated that ambiguity may have existed in either the advertisement itself or the response options. Further analyses should be undertaken to examine whether this item should be eliminated or revised. In addition, it was observed that the distribution of respondents’ ChMLM total scores was negatively skewed. Nevertheless, the ceiling effect is less of a concern because the high scores could be due to our relatively young and highly educated study sample. The difficulty levels of the ChMLM items should be adequate and discriminative for the general adult population in Taiwan.

The developed ChMLM can serve as a screening tool to identify those individuals with inadequate ML who may need additional assistance or intervention. For example, pharmacists and clinicians could use simpler terms, pictorial aids^39^ and/or visual medication schedules^40^ when educating or counselling these patients to enhance their understanding of medication‐related materials. In addition, the ChMLM could help pharmacists recognize the deficient areas where medication education needs more effort and improvement; the ultimate goal is to prevent potential errors or harm resulting from the misunderstanding of medication information.

The development and validation of the ChMLM is an essential starting point for ML research and investigation. This measure could be adapted for the use in any Chinese‐speaking population and country. As a next step, a national survey with a larger and representative sample needs to be conducted in Taiwan to investigate the ML levels of the general adult population and whether the ChMLM could be easily understood by lower educated population. Moreover, a larger sample would help confirm ChMLM's psychometric properties and also examine the factors associated with patients’ ML levels. In addition, to improve the ChMLM's practical use, future research is needed to develop and validate a short version of the ChMLM. The current full version takes around 15‐25 minutes to complete. A valid and reliable ML tool with a shorter completion time would surely increase health professionals’ willingness to use it.

This study has a few limitations. First, due to the concern of response burden, the type of medication props and the number of questions included in the ChMLM were limited. As a result, not all domains of medication literacy were tested, including other dosage forms (eg, syringe, suppository, nasal drop) and the ability to calculate a correct dose by weight for children. Nevertheless, the present items were developed and selected by an expert panel because they were considered important and commonly encountered by patients. Second, despite the fact that participants were offered “don't know/unsure” as a response choice for each question, guessing may not have been completely eliminated, and consequently, some respondents’ ML levels may have been overestimated. Third, although efforts were made to recruit eligible adults with diverse sociodemographic characteristics, volunteer bias may have been present. It is likely that individuals who were willing to participate in the survey were more confident with their ML skills and/or more interested in medication‐related information than those who were not. Lastly, medication props, instead of actual medication labels or packages, were used, and it is unknown whether respondents’ comprehension and behaviours would differed with actual materials.

## 5 CONCLUSION

This study demonstrated that the developed ChMLM is a valid and reliable performance‐based ML measure. It may help pharmacists and other health‐care providers to target patients and problem areas that need interventions with the ultimate goal of preventing medication errors and harm. The practical use of the ChMLM needs to be further examined in a representative sample.
